# Chromosomal microarray analysis versus noninvasive prenatal testing in fetuses with increased nuchal translucency

**DOI:** 10.1038/s10038-022-01041-0

**Published:** 2022-05-17

**Authors:** Chaohong Wang, Junxiang Tang, Keting Tong, Daoqi Huang, Huayu Tu, Jiansheng Zhu

**Affiliations:** Maternity and Child Health Hospital of Anhui Province, Affiliated Maternity and Child Health Hospital of Anhui Medical University, Hefei, China

**Keywords:** Genetics research, Chromosome abnormality

## Abstract

**Objective:**

To evaluate if the NT value of 2.5 mm ≤ NT < 3.0 mm is an appropriate indication for CMA tests among fetuses with isolated increased NT and NIPT is more suitable instead.

**Methods:**

A total of 442 fetuses with NT ≥ 2.5 mm were included, in which 241 fetuses underwent karyotype. CMA tests were then carried out when cytogenic analysis showed normal chromosomes and CNV status was compared between 2.5 mm ≤ NT < 3.0 mm and ≥3.0 mm subgroups. For the NIPT evaluation, 201 of 442 fetuses with smaller increased NT (2.5 mm ≤ NT < 3.0 mm) was examined by either NIPT or karyotype.

**Results:**

Of the 241 fetuses with NT ≥ 2.5 mm, 47(19.50%) were identified by karyotype with chromosomal abnormalities. Among 194 cases with normal karyotype, CMA unraveled additional CNVs in 16(8.25%) cases, including 3(1.55%) pathogenic CNVs, 2(1.03%) likely pathogenic CNVs and 11(5.67%) VOUS. After the subgroup analysis, however, only one case (1.16%) of likely pathogenic was identified by CMA among 86 fetuses with NT between 2.5 mm and 3.0 mm, whereas the rest of 15 CNV cases were all presented in fetuses with NT ≥ 3.0 mm. For the NIPT evaluation, the detection rate of 201 fetuses with isolated increased NT between 2.5 and 3.0 mm was 3.98%, which was indifferent to karyotype with the rate of 5%. In comparison with fetuses with 2.5–3.0 mm combined with other risks, the detection rate of karyotype was 26.92%.

**Conclusion:**

While no pathogenic CNVs were detected in fetuses, chromosomal aneuploidies and genomic imbalance were found to be the major type of abnormalities when NT was 2.5–3.0 mm. Therefore, our data suggested that CMA should not be recommended when fetuses with an NT value less than 3.0 mm. Instead, NIPT with similar rate of detection as karyotype was recommended for fetuses with isolated increased NT between 2.5 and 3.0 mm.

## Introduction

Nuchal translucency (NT) is the thickness of fluid collection in fetal neck, which could be observed by an ultrasound scan between 10 and 13 weeks of gestation. Previous publications demonstrated that fetuses with increased NT are at high risk of chromosomal abnormalities [1], fetal structure defects [2] and other pathological conditions, including congenital heart disease [3], infection in utero [4] and genetic syndromes [5]. More than 90% of fetuses with the major aneuploidies have been identified by measuring fetus NT value during the first trimester at a false positive rate of 5% in combination with maternal serum markers [6]. Therefore, first-trimester NT value is an important indication in prenatal diagnosis, which has been widely used clinically. Up till now, more than one hundred of diseases have been confirmed to be related to increased NT, whereas such association remain vague and more diseases are expected to be discovered in future.

Though karyotyping has been the gold standard prenatal diagnostic method for detection of chromosomal abnormalities, this technique has many limitations, such as labor intensive and longer laboratory turnaround time [7], higher failure rate for low quality and viability of samples [8]. Most important of all, smaller genomic imbalances including microdeletions and microduplications, which could be detected by chromosomal microarrays (CMA) are undetectable by karyotyping [9]. CMA is a high-resolution and high-throughput molecular detection technology for detecting human genomic DNA copy number variation, which shows advantages over karyotyping both in postnatal diagnosis and prenatal diagnosis. According to the most recent research, applying of CMA in fetuses with elevated NT identified additional pathogenic copy number variants (CNVs) over that from karyotyping in 3.7% [10]. However, no global consensus on the cut-off value of NT for CMA has been reached currently. Traditionally, most investigators have proposed using CMA for prenatal diagnosis, in cases with NT ≥ 3.5 mm [11–14]. Besides, 3.0 mm as the threshold value was also recommended for CMA detection in several reports [15, 16], in which additional pathogenic CNVs were detected in 5.12% of the fetuses. Most recently, Zhang et al. [17] who identified 5 pathogenic CNVs in cases with an NT of 2.5–3.5 mm and recommended CMA for the detection of pathogenic chromosomal aberrations in fetuses with an NT ≥ 2.5 mm. In addition, Su et al. [18] found that CMA significantly improved the detection rate of chromosomal abnormalities in fetuses with NTs of 2.5–3.4 mm and with normal karyotype, regardless of whether other ultrasonic abnormalities were observed. In spite of this, more data are necessary to elucidated whether NT cut-off value of 2.5 mm should be used as an indication for CMA.

The objective of this study was to evaluate the value of CMA in examining the genomic imbalances in fetuses with increased nuchal translucency (NT ≥ 2.5 mm) but normal karyotype and determine whether NT of 2.5 mm should be recommended as indication for CMA. In addition, the efficiency of NIPT in fetuses with NTs of 2.5–2.9 mm was also determined by comparing with karyotyping.

## Materials and methods

### Patients and samples

This was a retrospective study, in which 442 pregnant women with increased NT (NT ≥ 2.5 mm) were enrolled at the Prenatal Diagnosis Center, Maternity and Child Health Hospital of Anhui Province between January 2018 and October 2020. In this study, women with singleton pregnancies identified to have fetal NT exceeding 2.5 mm in thickness were considered eligible for inclusion, while fetuses with NT < 2.5 mm or those with any other findings on ultrasound were excluded. The maternal age was range from 17 to 44 years old and the gestational age was range from 12 to 23 weeks. Informed consent for genetic studies was obtained from all pregnant women, then participants were offered invasive testing. Amniotic fluid (AF) was obtained from pregnant women with the initial 2 ml of AF abandoned to avoid maternal cell contamination. G-banded karyotyping that is capable of detecting chromosomal abnormalities was used as the first step in searching for aneuploidies. Maternal peripheral blood samples (5 mL) were collected in EDTA tubes, fully mixed, stored temporarily at 4 °C and underwent NIPT. To evaluate the detection efficacy of NIPT in fetuses with smaller increased NT, the cohort of 287 fetuses with NT between 2.5 and 3.0 mm were grouped in this study. Of 261 fetuses with isolated NT, 60 fetuses underwent amniocentesis, while 201 cases preferred NIPT. 26 fetuses with smaller increased NT combined with other risks underwent amniocentesis.

### Conventional G-banded karyotyping

With the guidance of ultrasound, AF samples were collected from pregnant women and then cultured. G (Giemsa) banded metaphase microscopic images were captured since at the metaphase stage of cell division, chromosomes were clearly visible. According to the international system for human cytogenetic nomenclature (ISCN) 2016 [19], at least 30 metaphases were analyzed for each case, using an AI chromosome image analysis system (CytoVision, Switzerland), and 5 karyotypes were analyzed. When the chromosomal analysis was normal, the pregnant women was then consulted for CMA analysis. As for cases with structural chromosomal aberrations detected by karyotype analysis, CMA was also performed to explore the function of structure variation.

### Chromosomal microarray analysis

Genomic DNA was extracted from uncontaminated AF samples for CMA analysis using a Qiagen DNA Mini kit (250; Qiagen, Valencia, CA, USA), according to the manufacturer’s protocol. DNA samples were purified and concentrated using a NanoDrop 2000c spectrophotometer and Biophotometer plus (Eppendorf Inc.). For single nucleotide polymorphism (SNP) array analysis, the DNA was screened according to instruction of CytoScan 750 K arrays (Thermo Fisher, MA, USA) with the average inter probe distance of 100 kb. The data were collected by GeneChip^TM^ Scanner 3000 system, and analyzed using Chromosome Analysis Suite software (Affymetrix, Inc., Santa Clara, CA, USA).

We categorized CNVs into five groups: pathogenic CNVs, likely pathogenic CNVs, variants of uncertain significance (VOUS), likely benign CNVs, and benign CNVs in accordance to the American College of Medical Genetics (ACMG) and publicly available CNV databases, including Database of Genomic Variants (DGV; http://projects.tcag.ca/variation/); Online Mendelian Inheritance in man(OMIM; http://www.omim.org) and ClinGen (https://www.clinicalgenome.org/). If VOUS were identified in AF samples, peripheral blood was collected from both parents and then the results were deciphered by trained analysts. CMA findings were also compared with findings that would be obtained by non-invasive prenatal testing (NIPT), which was not performed in the present study.

### NIPT with the BGISEQ-500 sequencing platform

DNA extraction, library construction, and sequencing were performed according to the standard protocol of Human Molecular Genetics Guidelines at Anhui Maternal and Child Health Care Hospital. Maternal plasma (200 μL) was used for cell-free fetal DNA extraction with a BGISP-300 (BGI, Shenzhen, China) and Nucleic Acid Extraction Kit (BGI, Shenzhen, China). After DNA extraction, the DNA was subjected to end repair by end repair enzymes under the following conditions: 37 °C for 10 min and 65 °C for 15 min, followed by adaptor ligation at 23 °C for 20 min with the label adaptor and ligase. After end repair and adaptor ligation, PCR was used to amplify the DNA to the desired concentration with the following cycle conditions: 98 °C for 2 min, followed by 12 cycles at 98 °C for 15 s, 56 °C for 15 s, and 72 °C for 30 s and a final extension at 72 °C for 5 min. The DNA amplification products were quantified using a Qubit® 2.0 (Life Tech, Invitrogen, USA) and QubitTM dsDNA HS Assay Kits (Life Tech, Invitrogen, USA); a concentration ≥2 ng/μL was regarded as a qualified standard. The volume was calculated according to the concentration of each sample, and each sample of the same mass was mixed by pooling. Double-stranded DNA was thermally denatured after pooling, and cyclic buffer and ligase were added for a cyclization reaction. The DNA circles were used to prepare DNBs by rolling circle replication (RCR). The concentration of DNBs was quantified by a Qubit® 2.0 using QubitTM ssDNA Assay Kits (Life Tech, Invitrogen, USA), and DNB concentrations in the range of 8–40 ng/μL were considered appropriate. The DNBs were loaded onto chips and sequenced using the BGISEQ-500 sequencing platform (BGI, Shenzhen, China). Any sample that failed to meet the quality control criteria was reported as detection failure by NIPT.

The sequence based on NGS was compared with the reference sequence map of the human genome, and the percentage of each chromosome was calculated with Illumina Sequencing Analysis Viewer1.9.1 software. Z values were used to evaluate the actual disease situation of the samples, as previously reported [20]. Interventional prenatal diagnosis was recommended for high-risk pregnant women with NIPT, and the pregnancy out comes of all cases were followed up.

### Statistical analysis

SPSS statistical software (version 22, SPSS Inc., Chicago, IL, USA) was used for statistical analysis. Chi-squared tests were used to determine the differences between groups, results with *p* values of less than 0.05 were considered statistically significant.

## Results

**Fig. 1:**
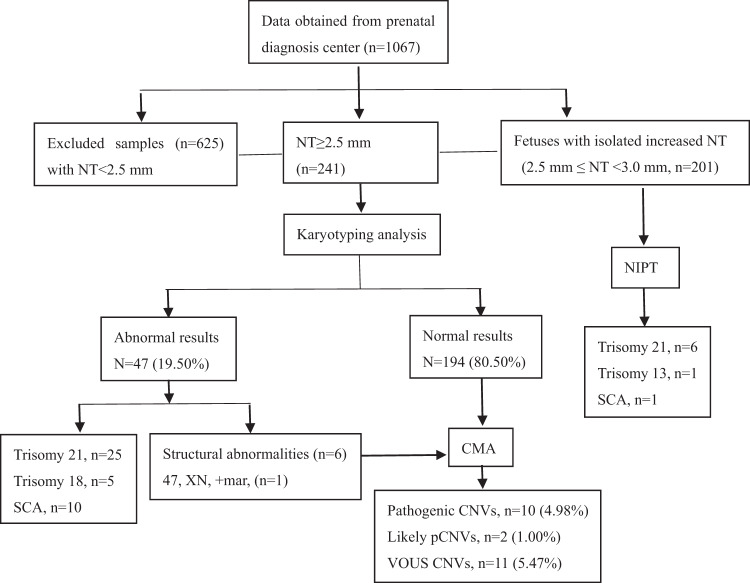
Flowchart of patient characteristics and chromosomal findings from karyotyping and CMA of 241 amniotic fluid samples from pregnancies with NT ≥ 2.5 mm

Figure 1 summarizes the patient characteristics and chromosomal findings detected by karyotyping and CMA. In one cohort of this study, 866 fetuses were grouped, of these, 241 pregnancies with increased fetal nuchal translucency (NT ≥ 2.5 mm) underwent invasive fetal karyotyping test. The NT value ranged from 2.5 mm to 9.4 mm, with a median of 3.5 mm. Of the 241 fetuses, 47 (19.50%) were identified by karyotype with chromosomal anomalies, including 25 cases of trisomy 21, 5 cases of trisomy 18, 10 cases of sex chromosomal aneuploidies (SCA) and 6 cases of chromosomal genomic imbalance as shown in Table 1. In 86 cases, the NT was 2.5–3.0 mm, and in 73 cases, the NT was 3.0–3.4 mm; while in 82 cases, it was greater than 3.5 mm. The median NT value of 47 cases with chromosomal abnormalities was 4.24 mm which was higher than that of 194 normal cases with a median NT value of 3.36 mm (*p* < 0.05).

**Table 1:** Chromosomal findings in fetuses with increased nuchal translucency thick (NT)

	NT(mm)						Total
	Karyotype	2.5–2.9	3.0–3.4	3.5–4.4	4.5–5.4	≥ 5.5	
Total		86	73	50	21	11	241
Normal	46,XN	76	62	41	11	4	194
Aneuploidy	47,XN,+21	4	5	4	8	4	25
	47,XN,+18	0	2	2	0	1	5
	47,XN,+mar	1	0	0	0	0	1
	SCA	3	2	3	1	1	10
Structural abnormality	46,XN,der(18)add(18)(p11.3)	0	1	0	0	0	1
	46,XN,der(18)add(18)(p11.2)	0	0	0	0	1	1
	46,XN,der(2)add(2)(q37)	0	1	0	0	0	1
	46,XN,del(11)(p12)	1	0	0	0	0	1
	46,XN,der(9)add(9)(p23)	1	0	0	0	0	1
	46,XN,der(5)add(5)(p15.3)	0	0	0	1	0	1
Detection rate (%)		11.63	15.07	18	47.62	63.64	19.50

A total of 194 cases with normal cytogenetic results and were then subjected to CMA analysis. As shown in Table 2, CMA unraveled 16 CNVs, including 3 pathogenic CNVs, 2 likely pathogenic CNVs and 11 cases of VOUS. The median NT value of 16 cases with CNVs was 3.69 mm which was also higher than that of 178 normal cases with a median NT value of 3.32 mm, though the difference between two groups did not reach statistical significance (*p* > 0.05). The distribution of categories of CNVs only detected by CMA was presented in Table 2 basing on NT thickness, the NT thickness thresholds used correspond to those used by Zhang et al. [17]. Only one (1/194, 0.52%) likely pathogenic CNVs were identified among the fetuses with NT value between 2.5 and 3.0 mm, two pathogenic CNVs (2/194, 1.03%) among the fetuses with NT value between 3.0 and 3.5 mm, and two (2/194, 1.03%) among the fetuses with NT value greater than 3.5 mm.

**Table 2:** Copy number variant (CNV) detection rates according to nuchal translucency thick (NT) in euploid fetuses

NT (mm)	*N* (%)	CMA				Detection rate (%)	χ2	*P*
		Pathogenic CNVs	Likely pathogenic CNVs	VOUS CNVs	Total			
2.5–2.9	76	0	1	6	7	1.32 (1/76)		
3.0–3.4	62	2	0	3	5	3.23 (2/62)	0.586	0.424
3.5–4.4	41	1	1	1	3	4.88 (2/41)	1.353	0.281
4.5–5.4	11	0	0	0	0	0 (0/11)	0.146	0.874
≥5.5	4	0	0	1	1	0 (0/4)	0.053	0.950
Total	194	3 (1.55%)	2 (1.03%)	11 (5.67%)	16			

All CNVs unraveled by CMA and karyotype analysis were relevant to clinically anomalies. The information of CNVs was shown in Table 3, seven cases had ≥10 Mb pathogenic CNVs including a 47,XN,+mar with a 14.11 Mb duplication on 10p12.1p11.1 (case 4) and 46,XN,del(11)(p12) of 14.79 Mb (case 12), which contained the WAGR syndrome critical region, one 5p15.33 deletion associated with Cri du Chat Syndrome (case 17) and other four cases (case 2, 3, 5, 18) of duplication and deletion syndrome with the size of genomic imbalance ranging from 14.11 Mb and 53.15 Mb. As for three pathogenic CNVs smaller than 10 Mb, one case was identified with 22q11.21 duplication syndrome (case 16), and one case with 1.4 Mb deletion located in 17p12 contains 5 genes (case 8). The other one with 5.37 Mb deletion in 9q22.32q31.1 may be associated with epileptic encephalopathy, language development disorder, macrosomia, learning disability and nephroblastoma (case 23). Termination of pregnancy was chosen by 8 of the 10 (80%) cases which were identified with pathogenic CNVs, while the other two women continued the pregnancy and ultimately gave birth to healthy babies.

**Table 3:** Details of copy number variant identified by CMA in fetuses with increased NT

Case	NT(mm)	CNV position(Human GRCh37/hg19)	Size(Mb)	Genes affected/syndromes	Categorization	Pregnancy outcome
1	3.0	4q35.1q35.2(186,167,916-187,842,570)x3	1.67 Mb dup	10 OMIM genes	VOUS	Unknown
2	3.3	7q31.31q36.3(120,072,971-159,119,707)x3,	39.03 Mb dup,	7q31.31qter duplication syndrome,	Pathogenic,	Therapeutic abortion
		18p11.32p11.31(136,227-3,251,461)x1	3.11 Mb del	10 OMIM genes	VOUS	
3	6.4	18p11.32p11.23(136,227-7,216,195)x1,	7.08 Mb del,	18p deletion syndrome,	Pathogenic,	Therapeutic abortion
		18q11.2q23(24,858,577-78,013,728)x3	53.15 Mb dup	18q11.2q23 duplication syndrome	Pathogenic,	
4	2.9	10p15.3(100,047-1,745,277)x4,	1.64 Mb dup,	10 OMIM genes,	VOUS,	Therapeutic abortion
		10p12.1p11.1(24,914,898-39,030,508)x2-3	14.11 Mb dup	43 OMIM genes	Pathogenic	
5	3.1	2q37.3(239,198,046-242,782,258)x1,	3.58 Mb del,	2q37.3 deletion syndrome,	Pathogenic,	Therapeutic abortion
		18q21.1q23(44,353,417-78,013,728)x3	33.66 Mb dup	89 OMIM genes	Pathogenic	
6	5.5	3q29(196,862,001-197,386,180)x3	524 Kb dup	2 OMIM genes	VOUS	Delivery, normal
7	2.8	15q13.2q13.3(31,098,690-32,915,723)x3	1.81 Mb dup	7 OMIM genes	VOUS	Delivery, normal
8	3.0	17p12(14,083,054-15,482,833)x1	1.4 Mb del	5 OMIM genes	Pathogenic	Delivery, normal
9	2.7	9p23(9,914,588-10,133,062)x1	218 Kb del	1 OMIM genes	VOUS	Delivery, normal
10	3.1	11q22.3(104,708,299-105,459,967)x3	752 Kb dup	7 OMIM genes	VOUS	Delivery, normal
11	2.8	1p13.2(112,802,599-113,868,278)x3	1.06 Mb dup	8 OMIM genes	VOUS	Delivery, normal
12	2.5	11p14.2p12(26,154,097-40,951,082)x1	14.79 Mb del	WAGR syndrome	Pathogenic	Therapeutic abortion
13	3.2	8p22(15,258,183-15,968,982)x3	711 Kb dup	2 OMIM genes	VOUS	Delivery, normal
14	2.9	2q37.3(241,490,065-242,782,258)x1	1.29 Mb del	25 OMIM genes	VOUS	Delivery, normal
15	3.9	6q12(65,196,218-65,743,530)x1	547 Kb del	1 OMIM genes	VOUS	Delivery, normal
16	3.3	22q11.21(18,970,561-21,461,017)x3	2.49 Mb dup	22q11.2 duplication syndrome	Pathogenic (nonpenetrance)	Delivery, normal
17	5.2	5p15.33p15.31(113,576-9,149,369)x1,	9.03 Mb del,	Cri du Chat Syndrome,	Pathogenic,	Therapeutic abortion
		5p15.31q11.1(9,153,500-49,475,697)x3	40.32 Mb dup	5p15.31q11.1 duplication syndrome	Pathogenic	
18	2.5	9p24.3p23(208,454-9,085,530)x1,	8.87 Mb del,	34 OMIM genes,	Pathogenic,	Therapeutic abortion
		13q31.1q34(87,397,574_115,107,733)x3	27.71 Mb dup	78 OMIM genes	Pathogenic	
19	2.8	12p11.21p11.1(32,407,341-34,897,417)x3,	2.49 Mb dup,	7 OMIM genes,	VOUS,	Delivery, normal
		12q11q12(37,856,237-42,720,825)x3	4.86 Mb dup	11 OMIM genes	VOUS	
20	2.7	1q21.1(145,382,123-145,775,966)x1	394 Kb del	14 OMIM genes	Likely pathogenic	Delivery, normal
21	4.4	8q22.3q23.1(103,348,066-108,445,788)x1	5.09 Mb del	17 OMIM genes	Likely pathogenic	Delivery, normal
22	2.9	22q11.21(20,716,876-21,464,764)x3	748 Kb dup	12 OMIM genes	VOUS	Delivery, normal
23	4.4	9q22.32q31.1(97,430,110-102,801,381)x1	5.37 Mb del	32 OMIM genes	Pathogenic	Therapeutic abortion

To evaluate the efficiency of noninvasive prenatal testing (NIPT) in detecting aneuploidies in fetuses with small increased NT, the detection rate of karyotyping and NIPT was compared in a cohort of 287 fetuses with NT between 2.5 and 3.0 mm. The results in Table 4 showed that one case of trisomy 21 and two cases of SCA were identified in 60 pregnancy women who preferred karyotyping and six cases of trisomy 21, one case of trisomy 13 and one case of SCA were identified in 201 pregnancy women who preferred NIPT with an detection rate of 5% and 3.98%, respectively. However, five cases of aneuploidy and two cases of structural abnormality were identified in the remaining 26 fetuses with combined risks, the detection rate was 26.92%.

**Table 4:** Comparison of detection rate of chromosomal anomalies by karyptype and NIPT in fetuses with 2.5 mm ≤ NT < 3.0 mm

Items	Result	NIPT		Karyotype
		Fetuses with isolated NT	Fetuses with isolated NT	Increased NT combined with other risks
Total		201	60	26
Normal	46,XN	193	57	19
Aneuploidy	47,XN,+21	6	1	3
	47,XN,+13	1	0	0
	47,XN,+mar	0	0	1
	SCA	1	2	1
Structural abnormality	46,XN,del(11)(p12)	0	0	1
	46,XN,der(9)add(9)(p23)	0	0	1
Detection rate (%)		3.98%	5%	26.92%
χ2			0.119	14.030
*P*			0.483	0.002

## Discussion

Increased NT was proved to be an important indication for chromosomal aneuploidies and fetal structure abnormalities [21, 22]. In the current study, 19.50% (47/241) of fetuses with NT ≥ 2.5 mm was identified with chromosomal aneuploidies, in which the trisomy 21 was the most common. This incidence was 32.29% as reported by Zhao et al. [16] and 12.2% by Zhang et al. [17], we ascribe these differences to various different NT cut-off values used among studies. These results also suggested that common aneuploidies in fetuses with increased NT could be diagnosed by conventional cytogenetic analysis instead of a further deep test. Though the American College of Obstetricians and Gynecologists (ACOG) recommended that the usefulness of CMA in detecting chromosomal abnormalities remains ambiguous and conventional karyotype analysis remains the preferred method of prenatal diagnostic testing [23]. In the past years, more and more studies have demonstrated advantages of CMA over karyotype analysis in prenatal diagnosis [10, 13]. In addition, a cut-off value of NT with 3.0 or 3.5 nm was used as the threshold in most investigations on the association between CMA and NT, the objective of this study was to determine whether CMA should be recommended on fetuses with NT ≥ 2.5 mm, but normal karyotypes.

In our cohort, 3 (1.55%) of fetuses with NT ≥ 2.5 mm but normal karyotypes were identified to be pathogenic genomic imbalance through CMA, which was comparable to the 1.4% in a study of 215 samples collected from the United Kingdom [24]. Egloff et al. [13] similarly reported 2% additional pathogenic CNVs by performing CMA in fetuses with isolated increased NT. On the contrary, this incidence was significantly lower than that reported by Zhang et al. [17], in which microarray detected additional pathogenic CNVs in 4.8% of fetuses with a NT greater than 2.5 mm. A meta-analysis based on 17 studies indicated that CMA provides approximately a 5.0% incremental yield for the detection of pathogenic CNVs above karyotyping [25]. Different detection rates among the studies might be associated with definition of “increased NT”, densities of probes, and cohort sample grouping. Of 86 cases with NT between 2.5 mm and 3.0 mm only one case (1.16%) was identified by CMA as likely pathogenic, and favorable pregnancy outcome was obtained according to follow-up information. Our results indicated that 2.5 mm might not be an appropriate threshold value in definition of “increased NT”.

In consistent with previous studies, 22q11.21 duplication syndrome was the most frequent clinically significant CNVs in our study, including a pathogenic CNVs (case 16) and a VOUS (case 22). It was reported that the 22q11.21 duplication syndrome was an extremely variable disorder with a phenotype, ranging from normal to learning disability, developmental retardation, hypotonia, growth restriction and intellectual disability [26, 27]. In spite of these risks, the pregnant woman preferred to keep pregnancy and had live birth without obvious developmental problem. The good pregnancy outcome might be associated with the incomplete penetrance of 22q11.21 duplication syndrome. Another pregnant woman with 1.4 Mb deletion in 17p12 who had normal ultrasonic examination also delivered healthy infant (case 8). The disadvantage was that follow-up of these cases were lasted for only months, there were no long-term outcomes available for infants in this study. Since some diseases were evident within years rather than at birth, long-term follow-up was necessary to ensure the outcomes of infants. The case (case 23) with variant in the 9q22.32q31.1 region showed ultrasonic anomalies, such as cervical fold thickening, bilateral cleft lip and palate, posterior fossa cystic structure and bilateral renal separation [28]. Finally, the pregnant woman chose termination of pregnancy.

Eleven cases (5.67%) were identified with VOUS in present study, which was higher than previous studies with rates ranging from 1 to 3% [29]. With proper consulting, all those VOUS cases prefer to continue pregnancy and all women gave birth to healthy infants except one case that was lost to follow-up. Since high proportion of VOUS cause too much inconvenience to pregnant women with anxieties and to doctors with significant challenges for genetic counseling. Thus, how to decrease the rate of VOUS will make sense in the future, and it is supposed that the rate of VOUS will be decreased with the improvement of local databases [16].

Of the 261 fetuses with isolated increased NT between 2.5 and 3.0 mm, 4.21% (11/261) were identified with chromosomal abnomalies in present study. But for the risk of amniocentesis, pregnancy woman with an isolated NT value less than 3.0 mm would not be recommended for karyotype analysis in clinical. Therefore, according to isolated increased NT between 2.5 and 3.0 mm, approximately 4.21% of fetuses with aneuploidy would have been missed. It is well known that NIPT screens maternal plasma for fetal aneuploidies by utilizing cell-free ‘fetal’ DNA originating from placental apoptosis, besides it has been widely accepted as an alternative to traditional invasive testing procedures [30, 31], though limited by not being able to identify majority of ‘atypical’ chromosomal anomalies reliably. Our result showed that for fetuses with isolated NT between 2.5 and 3.0 mm, the detection rate of NIPT was 3.98%, which was comparable to karyotyping with rate of 5%. No differences were observed in detection rate between NIPT and karyotyping in fetuses with isolated NTs of 2.5 and 3.0 mm. Whereas, 26.92% of fetuses with increased NT combined with other risks were diagnosed to be pathogenic. In previous study [32], CMA identified 3 aneuploidies in 86 fetuses with isolated increased NT (3.5%, 3/86), which was significantly lower than increased NT combined with other risks (39.5%, 32/81). The result suggested that fetuses with increased NT combined with other risks usually accompany with higher rate of chromosomal abnormalities than that with isolated increased NT. Our result is consistent with previous study [33], in which NIPT is acknowledged as an accurate approach for detection of multiple monogenic disorders among fetuses with skeletal abnormalities or increased NT with a positive result of 61.5% (8/13). In addition, NITP is also recommended as the first choice for fetal diagnosis in pregnant women with smaller increased NT who do not accept invasive prenatal diagnosis [34]. Inversely, Miranda et al. [14] suggested that NIPT does not appear to be the appropriate genetic test in fetuses with NT > 99th centile, given that it would miss 12–19% of genetic anomalies in their studies. We own the opposite conclusion to different NT cut-off value. The detection efficiency of NIPT in fetuses with smaller increased NT should be confirmed basing on large sample data in future. On all accounts, fetuses with increased NT combined with other risks should be recommended for invasive prenatal testing, while NIPT may be suitable for pregnancy woman with isolated NT between 2.5 and 3.0 mm.

## Conclusion

In conclusion, our study showed that most pathogenic CNVs could be identified by conventional karyotyping analysis, 1.5% of fetuses with NT ≥ 2.5 mm but normal karyotypes were identified to be pathogenic genomic imbalance by CMA, while no pathogenic CNVs were detected in fetuses when NT was 2.5–3.0 mm. Hence, we suggested that CMA should not be recommend when fetuses with smaller increased NT (2.5 mm ≤ NT < 3.0 mm). While no differences were observed in detection rate between NIPT and karyotyping in fetuses with isolated NTs of 2.5 and 3.0 mm. NIPT was recommended for fetuses with isolated increased NT between 2.5 and 3.0 mm.

## Data Availability

The data supporting the conclusions of this article is included within the article.
